# Does maternal diet during pregnancy and lactation affect allergy outcomes in their offspring? A systematic review of food based approaches

**DOI:** 10.1186/2045-7022-3-S3-O19

**Published:** 2013-07-25

**Authors:** M Netting, P Middleton, M Makrides

**Affiliations:** 1 Child Nutrition Research Centre, Women's and Children's Health Research Institute, North Adelaide, Australia; 2 ARCH: Australian Research Centre for Health of Women and Babies, The Robinson Institute, Discipline of Obstetrics & Gynaecology School of Paediatrics and Reproductive Health, The University of Adelaide, North Adelaide, Australia

## Background

Dietary strategies may prevent childhood allergies and reduce the burden of disease. We undertook this systematic review to evaluate the relationship between maternal diet and childhood allergies.

## Methods

We included studies published up to August 2011 which compared a food-based maternal dietary intervention (intake during pregnancy &/or lactation) with another intervention or no intervention, as well as studies examining associations between maternal dietary intake during pregnancy &/or lactation and allergic outcomes in their children from the index pregnancy. Primary outcomes included child eczema, asthma, hayfever and sensitization (to food or environmental allergens). Studies assessing dietary supplements were not eligible for inclusion, nor were studies where dietary intakes were expressed only in terms of nutrients. Studies exclusively investigating maternal nut and peanut consumption during pregnancy &/or lactation were excluded.

## Results

43 studies were included in this systematic review: 11 intervention studies, 27 prospective cohort studies, four retrospective cohort studies and one case-control study. In the RCTs no significant difference was noted overall in the prevalence of eczema and asthma in the offspring of women on diets free from common food allergens during pregnancy. One study (with multiple interventions) reported a lower rate of sensitisation in the intervention group at all ages followed up. Of the prospective cohorts only a few associations were reported between maternal dietary intake and development of allergy considering the overall number that were investigated. Of the results that were consistent, maternal dietary patterns were associated with less risk of allergic disease in offspring included ‘Mediterranean’ dietary patterns and diets rich in fruits and vegetables, fish and vitamin D containing foods. Foods associated with higher risk include vegetable oils and margarine, nuts and fast food. Studies differed in terms of the atopic potential of the participants and the final outcomes were not standardised in terms of age at follow up or diagnosis.

## Conclusion

The development of atopic disease is complex and multifactorial, depending on genetic potential along with many environmental influences. Maternal diet during pregnancy and lactation is one factor that may have the potential to influence the development of allergy in the child.

## Disclosure of interest

None declared.

